# Genome-Wide Detection of Copy Number Variations and Their Potential Association with Carcass and Meat Quality Traits in Pingliang Red Cattle

**DOI:** 10.3390/ijms25115626

**Published:** 2024-05-22

**Authors:** Yuanqing Wang, Jun Ma, Jing Wang, Lupei Zhang, Lingyang Xu, Yan Chen, Bo Zhu, Zezhao Wang, Huijiang Gao, Junya Li, Xue Gao

**Affiliations:** Laboratory of Molecular Biology and Bovine Breeding, Institute of Animal Sciences, Chinese Academy of Agricultural Sciences, Beijing 100193, China

**Keywords:** copy number variation, carcass and meat quality traits, Pingliang red cattle, candidate genes

## Abstract

Copy number variation (CNV) serves as a significant source of genetic diversity in mammals and exerts substantial effects on various complex traits. Pingliang red cattle, an outstanding indigenous resource in China, possess remarkable breeding value attributed to their tender meat and superior marbling quality. However, the genetic mechanisms influencing carcass and meat quality traits in Pingliang red cattle are not well understood. We generated a comprehensive genome-wide CNV map for Pingliang red cattle using the GGP Bovine 100K SNP chip. A total of 755 copy number variable regions (CNVRs) spanning 81.03 Mb were identified, accounting for approximately 3.24% of the bovine autosomal genome. Among these, we discovered 270 potentially breed-specific CNVRs in Pingliang red cattle, including 143 gains, 73 losses, and 54 mixed events. Functional annotation analysis revealed significant associations between these specific CNVRs and important traits such as carcass and meat quality, reproduction, exterior traits, growth traits, and health traits. Additionally, our network and transcriptome analysis highlighted *CACNA2D1*, *CYLD*, *UBXN2B*, *TG*, *NADK*, and *ITGA9* as promising candidate genes associated with carcass weight and intramuscular fat deposition. The current study presents a genome-wide CNV map in Pingliang red cattle, highlighting breed-specific CNVRs, and transcriptome findings provide valuable insights into the underlying genetic characteristics of Pingliang red cattle. These results offer potential avenues for enhancing meat quality through a targeted breeding program.

## 1. Introduction

Marbling plays a critical role in determining the quality of meat, significantly contributing to its sensory characteristics, with consumers often considering the degree of marbling as the primary factor influencing their purchasing decisions. Therefore, understanding the driving factors that influence marbling pattern can enhance meat quality and yield greater economic benefits. Copy number variations (CNVs) are key structural variations in which DNA segments ranging from 1 kilobase to several megabases undergo duplication or deletion, thereby leading to substantial genetic variation [1,2]. In terms of the total number of bases involved, CNVs encompass a large number of nucleotide sequences and occur more frequently than SNPs [3,4]. Consequently, they have a higher probability of mutation and more significant potential impacts, such as altering gene structure, modifying gene dosage, and consequently dramatically affecting gene expression and adaptive phenotypes [5]. There are four commonly used methods for detecting CNVs, including comparative genomic hybridization arrays (CGH array), SNP arrays (Illumina BovineHD BeadChip, Illumina BovineSNP50 BeadChip), short-read next-generation sequencing (NGS), and single-molecule long-read sequencing (SMRT) methods [6]. Indeed, multiple studies have suggested that CNVs play an essential role in the development of some complex traits and disease [7]. Research on CNVs in humans has revealed their association with phenotypic diversity and complex diseases such as rheumatoid arthritis, cancer, and developmental delay.

A substantial number of studies have been widely conducted to analyze genome-wide CNVs of domestic animals using high-throughput and high-density technologies, which enable CNVs to serve as crucial molecular markers for complex traits in genome-wide association analysis studies (GWASs) [8]. Silva et al. discovered that genes within CNVRs in the Nellore cattle genome are involved in guanosine triphosphate (GTP) biological processes. Through genome-wide association analysis, they also found that cGMP and cAMP pathway genes are located within genomic regions significantly associated with meat tenderness [9]. In another study analyzing the association between CNVRs in Nellore cattle and beef fatty acids, researchers identified 186 CNVRs that are significantly correlated with saturated fatty acids (SFAs), monounsaturated fatty acids (MUFAs), polyunsaturated fatty acids (PUFAs), and omega fatty acid groups [10]. Zhang et al. discovered a relationship between the CNVs of the *GBP2* gene and growth traits using array-based comparative genomic hybridization (aCGH) [11]. Yue et al. investigated the CNVs of the bovine *HSFY* and *ZNF280BY* in a sample of 460 bulls across 15 breeds. The findings indicated a negative correlation between the CNVs of *HSFY* and *ZNF280BY* with testis size, while displaying a positive correlation with sire conception rate. The analysis of SNP genotype data among 472 Angus cattle resulted in the detection of 811 potential CNVRs. By comparing the resistance to parasites and susceptibility among individuals, it was observed that certain CNVs were associated with variations in host parasite resistance levels [12]. Sun et al. identified a set of positive selected genes (PSGs) and CNVRs overlapped with quantitative trait loci (QTLs) involved in milk, immunity, and reproduction by combining the high-coverage short-read NGS data and SMRT data. These findings indicate that CNVs in genes can have important influences on the economic characteristics of domestic animals and enrich organismal diversity.

Pingliang red cattle (PRC), a native species from Gansu Province in China, exhibits an exceptional breeding value, which is attributed to its tender meat and superior marbling quality. However, limited research has been conducted on detecting CNVs and analyzing their association with carcass and meat quality traits in PRC. Therefore, the objective of this study was to detect CNVs in the genome of Pingliang red cattle to gain a comprehensive understanding of the genetic mechanisms underlying population-specific traits. Genome-wide CNV detection was carried out using GGP Bovine 100K SNP genotype data from 122 Pingliang red cattle. Comparative analysis with previous studies allowed us to identify CNVRs and candidate genes associated with carcass meat quality traits, which could serve as potential genetic markers for selecting desirable meat quality traits in Pingliang red cattle.

## 2. Materials and Methods

### 2.1. Sample Collection and Genotyping

To ensure the representativeness of the experimental samples, efforts were made to collect the samples from genetically unrelated individuals based on pedigree records and information provided by farmers. A total of 122 blood samples were collected from Pingliang red cattle breeding farms in seven counties and districts of Pingliang City, Gansu Province, China. For SNP-array analysis, genomic DNA samples were extracted from blood and genotyped using the GGP Bovine 100K SNP chip, which included a total of 95,256 SNPs and an average SNP spacing of 29.0 kb.

### 2.2. Transcriptome Sequencing

Samples for transcriptome analysis were collected from a Dingkang beef cattle fattening farm in Jingchuan County, Gansu Province, China. The 10 unrelated steers (Pingliang red cattle) were raised under identical feeding and management conditions. At the age of 3 years and a weight of 780.0 ± 39.2 kg, the animals were transferred for slaughter 8 km away to Xukang Food Co., Ltd., Jingchuan County. Five steers were sampled at each slaughter time, with a time interval of 4 days. After slaughter, 2 cm^2^ pieces of the internal section of the longissimus lumborum, triceps brachii, liver, and back fat from each animal, isolated immediately, and immersed in liquid nitrogen (−80 °C) for RNA extraction.

Total RNA was isolated from the 40 samples using TRIzol reagent following the manufacturer’s recommendations (Invitrogen, Life Technologies). RNA concentration and purity were determined using a Qubit 2.0 Fluorometer and NanoDrop spectrophotometer (Thermo Fisher Scientific, Waltham, MA, USA). First-strand cDNA was synthesized using a random hexamer primer and M-MuLV Reverse. Second-strand cDNA synthesis was subsequently performed using DNA Polymerase I and RNase H. The cDNA fragments, which were 250–300 bp in length, were purified preferentially with the AMPure XP system. Finally, raw data were generated on the DNBSEQ-T7 sequencing platform (150 bp paired-end reads) (Beijing Genomics Institute, Shenzhen, China).

### 2.3. Genome-Wide CNV Detection and Quality Control

The PennCNV v1.0.5 software was utilized to identify the CNVs in the genome of Pingliang red cattle. The GenomeStudio v2011.1 program was used to generate the input signal intensity file, which contained essential information required for CNV calling, including the SNP name, chromosome, position, genotype, Log R Ratio (LRR), and B Allele Frequency (BAF). The PFB file was generated by running the perl script “compile_pfb.pl” provided by the PennCNV program based on the average BAF of each marker in the population. To eliminate the impact of genomic waves on the CNV calling procedure, the GC model file, which was generated by calculating the 1 Mb genomic regions surrounding each marker (500 K on each side), was used as a parameter of option “-gcmodel”. After preparing all the necessary files, the script “detect_cnv.pl” was used to identify CNVs in all individuals. Finally, quality control measures were applied using cutoff values (LRR standard deviation 0.30, BAF drift 0.01, and waviness factor 0.05) with the function “filter_cnv.pl”. Altogether, 5 individuals were excluded from subsequent analysis; thus, a total of 117 were included.

### 2.4. CNV Compilation

The CNVRuler software v1.3.3.2 was used to merge CNVs across all samples, requiring a minimum of 1 bp overlap in order to define the CNVRs. The “Gain/Loss separated regions” option was employed to compile the CNVRs based on their genotypes, resulting in three distinct types: loss, gain, and mixed (both gains and losses occurred within the same region). In addition, considering that copy number variations are defined as genomic segments ranging from 1 kb to several Mbs with variable copy numbers compared to the reference genome, any CNVRs smaller than 1 kb were excluded from the analysis [13]. To visually represent these CNVRs on a chromosome diagram, HandyCNV (R Packages) was used by assigning different colors for “Gain”, “Loss”, and “Mixed” [14].

### 2.5. CNVR Annotation and Enrichment Analysis

The genes harbored within or partially overlapping with CNVRs were retrieved from the UCSC genome browser (ARS-UCD 1.2) using BioMart (http://www.biomart.org/) (accessed on 12 May 2022). Additionally, a gene set enrichment analysis of the candidate genes was also conducted using DAVID v6.8 (https://david.ncifcrf.gov/summary.jsp) (accessed on 12 May 2022). To investigate the potential correlation between CNVRs and important economic traits in cattle, gene annotation was further performed using the Cattle QTL database.

### 2.6. Comparison with Other Studies of CNVRs in Cattle

In order to validate the accuracy of the detected CNVRs and identify specific CNVRs in Pingliang red cattle, we compared our results with those from previous studies involving various breeds of cattle, yak, buffalo, and indicine. Since some of these previous results were mapped on the Btau 4.0 and UMD 3.1 genome builds, we converted those results from Btau 4.0 or UMD 3.1 to ARS-UCD 1.2 using the LiftOver tool (https://genome.ucsc.edu/cgi-bin/hgLiftOver) (accessed on 12 May 2022) on UCSC before comparison. The CNVRs on non-autosomal chromosomes or in unplaced locations were also excluded before analysis. Overlaps between the results obtained in the present study and those from previous reports were evaluated based on a criterion requiring an overlap of at least 1 bp [15]. Subsequently, functional enrichment analyses and QTL annotation were carried out on CNVRs that were deemed specific to Pingliang red cattle.

### 2.7. Validation of Candidate Genes Using Transcriptome Analysis

To validate the candidate genes, a network diagram was firstly drawn using Cytoscape software (v.3.9.1) to explore the interaction of the candidate genes [16]. Then, we performed further analysis using transcriptome sequencing data. The fastp tool (v.0.19.7) was applied to remove paired-end reads from the raw data in line with the following aspects: (1) any read with N content exceeding 10% of the read bases; (2) any read with more than 50% low-quality bases (Q ≤ 5); and (3) any read containing adapter remnants [17]. SAMtools (v.1.13) was used for sorting and format conversion of SAM files [18]. And we applied the feature Counts (v.2.0.0) and StringTie (v.2.1.7) programs to compute the read count and the fragments per kilobase of exon per million mapped reads (FPKMs) per sample, respectively [19,20]. The expression levels of candidate genes were visualized using the Pheatmap (R packages).

## 3. Results

### 3.1. Genome-Wide Detection of CNVs and CNVRs

**Figure 1:**
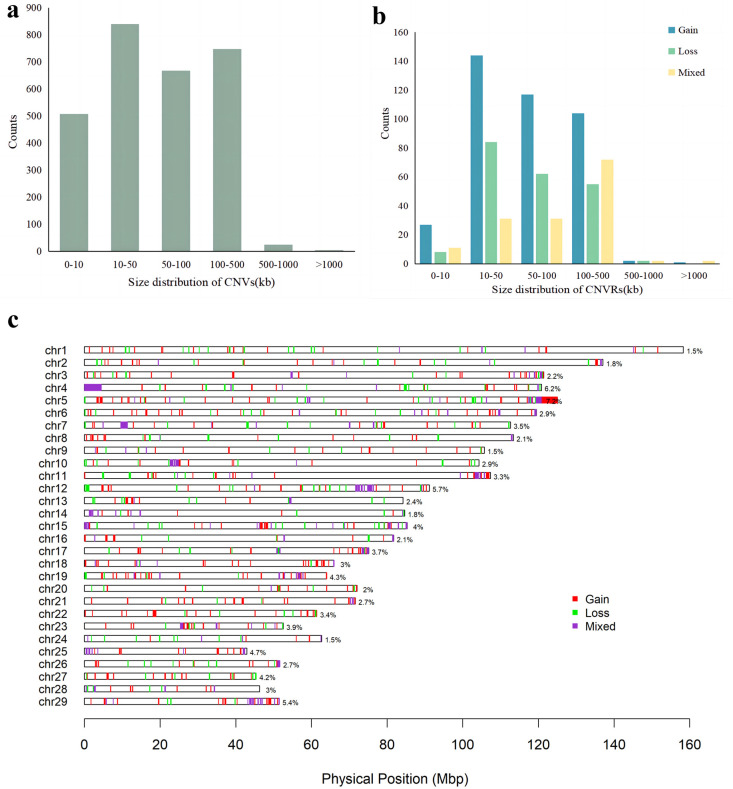
Identification of copy number variation (CNV) in Pingliang red cattle. (**a**) Size distribution of CNVs. (**b**) Size distribution of CNVRs. (**c**) Whole-genome CNVR map. Red, green, and violet represent gain events, loss events, and mixed events, respectively.

We conducted CNV analysis on the genome of Pingliang red cattle using the GGP Bovine 100K SNP chip and PennCNV software v1.0.5. A total of 2793 CNVs were detected in 117 individuals after quality control. These autosomal CNVs had a mean size of 89.19 kb, with a median length of 52.61 kb. Approximately 30.08% of the CNVs ranged from 10 kb to 50 kb, while 23.88% were between 50 kb and 100 kb, and another 26.78% ranged from 100 kb to 500 kb; small-segment CNVs accounted for 18.19%, measuring less than 10 kb (Table S1 and Figure 1a).

After aggregating the overlapping CNVs, we identified a total of 755 CNVRs across the autosomal genome, with a total length of 81.03 Mb, covering 3.24% of the entire genome. The majority (92.71%) of these CNVRs ranged from 10 bp to 500 kb in size, while larger ones (>500 kb) were rarely observed (1.19%). Overall, CNVRs ranged in size from 1.01 kb to 4.44 Mb with an average size of 107.32 kb (Table S1 and Figure 1b).

Among the 755 identified CNVRs, 395 were gain events, 211 were loss events, and 149 CNVRs were composed of both gain and loss events. Distributions of autosomal CNVRs are presented in Table S2 and Figure 1c. The proportion of coverage by CNVRs varied among chromosomes, ranging from 1.45% to 7.24% (ARS-UCD 1.2). The number of CNVRs on each chromosome also differed, ranging from 14 on BTA28 to 55 on BTA5. The closest interval between CNVRs in the genome was 3.87 kb on BTA6, and the largest interval was 49.08 kb on BTA11 (Figure 1c).

### 3.2. CNVR Annotation and Enrichment Analysis

The BioMart tool (R Packages) was employed to map genes to the 755 identified CNVRs. In total, 1219 genes that either completely or partially overlapped with CNVRs were identified. Among these genes, there were 1087 protein-coding genes (89.17%), 62 lncRNA genes (5.09%), and 24 miRNA genes (1.97%). The remaining genes consisted of pseudogenes, snoRNA genes, and snRNA genes (Table S3).

**Figure 2:**
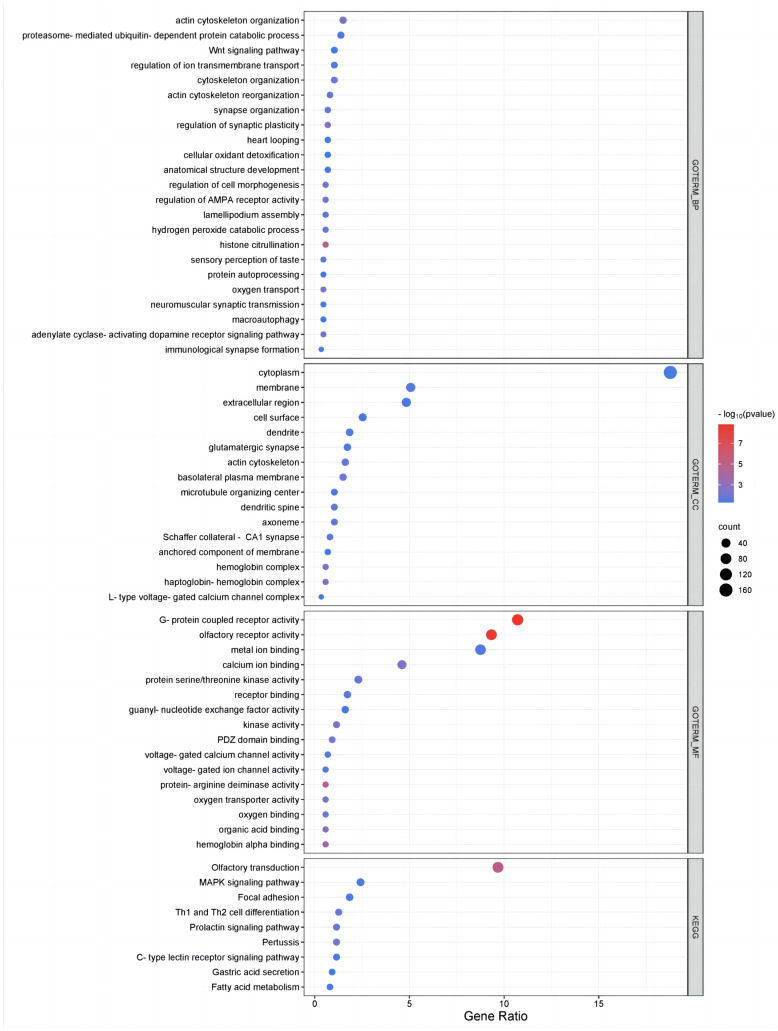
Functional enrichment analysis of CNVR-related genes in the genome in Pingliang red cattle.

To further investigate the biological functions of genes harbored in the different types of CNVRs in Pingliang red cattle, an enrichment analysis of protein-coding genes was conducted using the DAVID v6.8 tool. The results of our investigation revealed significant enrichment in 55 GO terms and 9 pathways (Table S4 and Figure 2). GO analyses revealed genes that play a dominant role in the CNVRs of Pingliang red cattle, including genes related to biological functions, such as histone citrullination (GO:0036414), actin cytoskeleton organization (GO:0030036), and oxygen transport (GO:0015671) in the BP category; the haptoglobin–hemoglobin complex (GO:0031838) and hemoglobin complex (GO:0005833) in the CC category; and G-protein-coupled receptor activity (GO:0004930) and olfactory receptor activity (GO:0004984) in the MF category. Additionally, the KEGG pathway analysis indicated that genes associated with CNVRs were primarily enriched in pathways such as olfactory transduction (bta04740), pertussis (bta05133), focal adhesion (bta04510), the MAPK signaling pathway (bta04010), and fatty acid metabolism (bta01212).

In addition, by integrating the analysis with data from the Cattle QTL database, the study identified 73 CNVR-associated genes that are related to important economic traits in cattle (Table S5). Among these genes, *CACNA2D1*, *CYLD*, *NADK*, *TG*, *CDH12*, *GALNT13*, *UBXN2B*, and *ITGA9* were found to be related to carcass and meat quality traits. Genes like *KCNIP4*, *PAK5*, *CYP21*, *CYLD*, *CCKBR*, *PCCA*, *PRSS2*, *MFGE8*, and *LRP5* are associated with growth traits. Additionally, *KCNIP4*, *MFGE8*, *PHACTR1*, *ABCC9*, *MACROD2*, *NPFFR2*, *PAK5*, *TBC1D24*, *TRAPPC9*, and *ACACA* were found to be linked to reproduction traits. These findings suggest that the CNVRs identified in our study may be associated with the genetic characteristics of Pingliang red cattle.

## 4. Comparison with Previous Studies of Bovine CNVRs

The findings from our comparison with 23 previously published bovine CNVR studies are summarized in Table S6. These earlier investigations reported varying numbers of CNVRs, ranging from 96 to 13,234, with lengths varying from 13.1 Mb to 498 Mb. The CNVRs that overlapped with our findings ranged from 2 to 208, with overlap percentages ranging from 0.26% to 27.55%. Overall, approximately 64.24% (485) of the CNVRs displayed varying degrees of overlap with those identified in previous studies, with a greater extent of overlap being observed when using the high-density chip (770 K), which ranged between 3.05% and 27.55%, as opposed to the lower range found using the SNP chip (50 K), which ranged between 0.26% and 6.62%.

Among the CNVRs identified using the 50K chip, a significant level of overlap was observed with the CNVRs detected in Holstein (859 individuals) and Polish Red cattle (301 individuals) by Gurgul et al. (2015). Specifically, 50 CNVRs (6.62%) were found to overlap, covering a combined length of approximately 5.99 Mb (7.40%) [21]. In contrast, when utilizing the high-density 770K chip, it was observed that a total of 208 CNVRs (27.55%) overlapped with CNVRs previously reported by Zhang et al. (2020), encompassing an overall length spanning around 27.99 Mb (34.55%) [22]. Furthermore, comparisons were conducted between our findings and those from CNV studies based on CGH arrays, resulting in overlaps ranging from as low as five up to seventy-three overlapping CNVRs, with cumulative lengths varying from 0.37 Mb to 16.46 Mb. Finally, our study also revealed overlaps between our CNVRs and those previously reported by Choi et al. (2013), Bickhart et al. (2012), Liu et al. (2019), Hu et al. (2020), and Gao et al. (2017) based on whole-genome resequencing data. The cumulative lengths for the overlapping regions were approximately 7.37 Mb (9.09%), 9.38 Mb (11.58%), 14.86 Mb (18.34%), 28.93 Mb (35.70%), and 29.36 Mb (36.24%), respectively [23,24,25,26,27].

A total of 270 CNVRs were identified as potentially specific to Pingliang red cattle, excluding those reported in the 23 previous studies. These specific CNVRs consisted of 143 gains, 73 losses, and 54 mixed events, encompassing a total of 200 genes. Functional annotation and enrichment analyses revealed significant enrichment in various biological functions, such as ATP binding (GO:0005524), neuronal cell bodies (GO:0043025), the rhythmic process (GO:0048511), negative regulation of microtubule depolymerization (GO:0007026), cell adhesion (GO:0007155), actin cytoskeleton (GO:0015629), and sensory perception of sour taste (GO:0050915). Additionally, KEGG analysis showed that the significantly enriched pathways included tight junction (bta04530) and focal adhesion (bta04510). Moreover, integrating data from the Cattle QTL database revealed that the genes associated with the Pingliang red cattle-specific CNVRs were those linked to carcass and meat quality, reproduction, exterior traits, growth traits, and health traits. These genes include *UBXN2B*, which was associated with carcass weight; *ITGA9*, *SEMA5AA*, and *GALNT1*3, which were associated with body fat deposition performance; *KCNIP4*, *MFGE8*, and *ACACA*, which were linked to calving ease and the reproductive index; and *KCNIP4* and *CYLD*, which were related to body height, weaning weight, and yearling weight.

### The Interaction and Different Expression Analysis of Candidate Genes in Four Tissues

**Figure 3:**
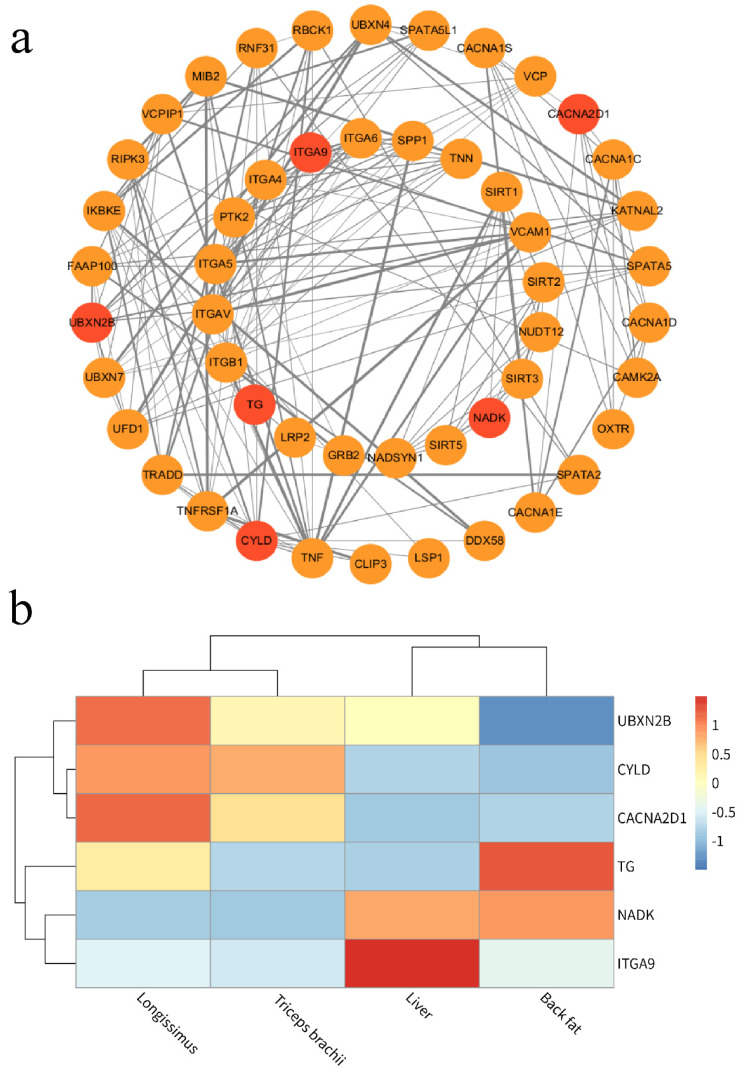
The network and heatmap of candidate genes for carcass and meat quality traits in Pingliang red cattle. (**a**) The network was built from known protein interactions (edges) between gene products (the red circles represent the key genes we identified). (**b**) The heatmap of 6 candidate genes involved in carcass and meat quality trait in four tissues.

Given that the emphasis of the present study was on investigating the candidate genes of the carcass and meat quality traits, we used the Cytoscape software to construct a personalized pathway diagram illustrating the identification of candidate genes based on annotation results (Figure 3a). Remarkably, *ITGA9*, *CACNA2D1*, *CYLD*, *NADK*, *TG*, and *UBXN2B* were found to be located at node positions within a metabolism pathway and exhibited a significant association with meat quality traits. This suggests that these genes potentially play a regulatory role in carcass and meat quality, and so we identified them as major candidate genes.

To further assess the reliability of these candidate genes, we focused on analyzing the expression levels of these six candidate genes in four tissues (Figure 3b). Two genes (*TG* and *NADK*) exhibited significantly higher expression in back fat compared to the other three tissues, while the remaining genes showed lower expression levels. Certain genes demonstrated elevated expression in longissimus lumborum (*CACNA2D1*, *UBXN2N*, and *CYLD*), triceps brachii (*CYLD*), and the liver (*ITGA9* and *NADK*). The divergent expression levels of these genes may have an impact on marbling quality and carcass weight across different beef tissues. The divergent expression levels of these genes may have an impact on marbling quality and carcass weight across different beef tissues.

## 5. Discussion

Copy number variation is increasingly recognized as a significant source of genetic variation and a key contributor to phenotypic diversity and evolutionary adaptation in animals. Previous research has demonstrated the influence of CNVs on various economically important traits in livestock, including growth traits [28], meat quality traits [10], milk production [29], disease resistance [12], and coat color [2]. The Pingliang red cattle is an excellent local breed with high intramuscular fat deposition. In this study, we used the GGP Bovine 100K SNP genotyping chip to generate a genome-wide CNV map of the Pingliang red cattle, aiming to uncover the association between the CNVRs in the genome and their outstanding breeding characteristics. By employing the PennCNV software for analysis, we detected a total of 755 CNVRs with a length exceeding 1kb on the autosomes of Pingliang red cattle. These CNVRs spanned a total length of 81.03 Mb, accounting for approximately 3.24% of the genome. Notably, there were significant disparities in CNVR content across different chromosomes, with the proportion of CNVRs to the total length of the chromosome ranging from 1.45% to 7.24%. Chromosomes 4, 5, and 12 had the longest total length of CNVRs.

It is worth noting that 64.24% of the detected CNVRs in this study showed varying degrees of overlap with those reported in previous studies. This also indicates that the CNVRs detected in this study have a high reliability. The degree of overlap varied among CNVRs identified in this study and those reported in prior studies using various platforms, such as CGH (0.66~9.67%), 50K (0.26~6.62%), 770K (3.05~27.55%), and resequencing (3.97~18.54%). Specifically, regarding previous studies utilizing SNP genotyping chips, the overlap between our study’s findings and those of the 770K high-density chip study tended to be higher compared to the findings of the study using the 50K SNP genotyping chip. This observation might be attributed to the number of overlapping SNP loci in the design of different chip models.

To further investigate the biological functions of genes harbored in these different types of CNVRs in Pingliang red cattle, we conducted an enrichment analysis. We found that the genes annotated by CNVRs in the genome of Pingliang red cattle are related to biological functions such as sensory perception of taste, oxygen transport, oxygen binding, and ion binding. It has been reported that many genes involved in sensory receptors, including olfactory receptors, are associated with CNVs [30], and olfactory-related genes have been discovered in previous CNV studies of various species, such as cattle [9], yaks [8], sheep [31], pigs [32], and others. Taste- and olfactory-related genes are crucial for organisms to adapt to the environment; their relevant CNVs may help them acquire food more effectively at night. Intramuscular fat content is an important indicator of muscle quality, and the composition of fatty acids in meat is the basis of meat flavor, which is important for human health [33]. KEGG pathway analysis also revealed that genes within CNVRs are significantly enriched in the fatty acid metabolism pathway (bta01212), including genes such as *CPT2*, *ELOVL5*, *TECR*, *PPT2*, *CPT1B*, *ACACA*, and *FADS1*. In addition, this study also found that genes associated with specific CNVRs in the genome of Pingliang red cattle are significantly enriched in functional annotations, such as sour taste perception, energy metabolism, and cell interaction, as well as biological pathways such as focal adhesion and tight junction. Focal adhesion (FA) is a sticky contact point between cells and the extracellular matrix. These complex structures regulate the communication between cells and the surrounding extracellular environment. Organisms regulate processes such as proliferation, migration, apoptosis, diffusion, and differentiation through FA [34].

In order to reveal the association between CNVRs in the genome of Pingliang red cattle and their germplasm characteristics, we utilized information from the Cattle QTL database regarding important economic traits QTL in cattle. Our analysis revealed that there were 73 genes within CNVRs that overlapped with QTL positions related to crucial economic traits, such as carcass meat quality, growth and development, reproduction, and immunity in cattle. Among these genes, 21 were specifically located within Pingliang red cattle-specific CNVRs. A personalized pathway diagram demonstrated that *CACNA2D1*, *CYLD*, *UBXN2B*, *TG*, *NADK*, and *ITGA9* occupied node positions within a metabolism pathway and exhibited significant associations with meat quality traits. Furthermore, transcriptome analysis showed that expression levels of *CACNA2D1*, *UBXN2N*, and *CYLD* were elevated in longissimus lumborum; *TG* and *NADK* displayed significantly higher expression in back fat, while *ITGA9* was highly expressed in liver. One prominent characteristic of Pingliang red cattle is their ability to produce high-quality marbled beef. The genes identified in this study that are associated with meat quality and carcass traits align with previous findings. Previous studies have indicated that TG is one of the candidate genes linked to intramuscular fat deposition levels and that its polymorphisms affect the marbling pattern of the longissimus dorsi in German Holstein and Charolais cattle [35]. The *ITGA9* gene encoding integrin subunit alpha 9 has been associated with fat thickness in Nellore cattle [36]. Another study revealed an association between *CACNA2D1* and fat thickness at the 12th rib as well as fat color and carcass weight [37]. Additionally, we discovered three genes (*CYLD*, *NADK*, and *UBXN2B*) associated with carcass weight that were previously reported in Hanwoo cattle within the CNV interval of the Pingliang red cattle genome [38,39].

## 6. Conclusions

Here, we generated a comprehensive genome-wide CNV map of Pingliang red cattle using GGP Bovine 100K SNP chip genotype data from 122 Pingliang red cattle. In total, 755 CNVRs were discovered in the Pingliang red cattle genome, with a total length of 81.03 Mb, comprising 3.24% of the bovine autosomal genome. Out of these, 270 CNVRs were newly identified as potentially specific to Pingliang red cattle, being linked to carcass and meat quality, reproduction, exterior traits, growth traits, and health traits. The results of our network and transcriptome analysis revealed *CACNA2D1*, *CYLD*, *UBXN2B*, *TG*, *NADK*, and *ITGA9* as promising candidate genes associated with carcass weight and intramuscular fat deposition. Our study provides valuable insights into the underlying genetic characteristics of Pingliang red cattle and offers potential avenues for improving meat quality in breeding programs.

## Data Availability

The genotypic data used and/or analyzed during the current study are available from the corresponding author on reasonable request.

## Supplementary Materials

The following supporting information can be downloaded at: https://www.mdpi.com/article/10.3390/ijms25115626/s1.

## Abbreviations

CNV: copy number variation; CNVR: copy number variation region; GWAS: genome-wide association studies; SFAs: saturated fatty acids; GTP: Guanosine triphosphate; aCGH: array-based comparative genome hybridization; MUFAs: monounsaturated fatty acids; PUFAs: polyunsaturated fatty acids; QTL: quantitative trait locus; PRC: Pingliang red cattle; GO: gene ontology; KEGG: Kyoto Encyclopedia of Genes and Genomes; BAF: B Allele Frequency; LRR: Log R Ratio; FA: focal adhesion.
