# Supplementary material for: Genome-Wide Detection of Copy Number Variations and Their Potential Association with Carcass and Meat Quality Traits in Pingliang Red Cattle
Source: Int J Mol Sci. 2024 May 22;25(11):5626. doi: 10.3390/ijms25115626 (PMC11172001; doi:10.3390/ijms25115626)
Supplement: Supplementary file 1 [file ijms-25-05626-s001.zip › ijms-3014312-supplementary.pdf]

Table S1. CNV and CNVR size distributions.

Size (kb)	Number of NVs	Percentage %	Number of CNVRs	Percentage%
0-10	508	18.19%	46	6.09%
10-50	840	30.08%	259	34.30%
50-100	667	23.88%	210	27.81%
100-500	748	26.78%	231	30.60%
500-1000	25	0.90%	6	0.79%
>1000	5	0.18%	3	0.40%

Table S2. The distribution of CNVRs in the Pingliang red cattle genome (based on UMD_3.1).

Chr	No.of CNVRs	Length of CNVRs (bp)	Average length (kb)	Percentage (%)
1	40	2,348.44	58.71	1.48%
2	29	2,531.72	87.30	1.86%
3	34	2,693.11	79.21	2.23%
4	30	7,488.78	249.63	6.24%
5	55	8,698.53	158.16	7.24%
6	44	3,489.52	79.31	2.96%
7	32	3,969.27	124.04	3.59%
8	21	2,396.41	114.11	2.11%
9	22	1,533.63	69.71	1.45%
10	22	3,070.59	139.57	2.97%
11	29	3,523.77	121.51	3.29%
12	39	5,235.44	134.24	6.00%
13	19	2,018.23	106.22	2.42%
14	19	1,512.37	79.60	1.84%
15	29	3,435.20	118.46	4.04%
16	16	1,746.86	109.18	2.16%
17	25	2,755.57	110.22	3.77%
18	26	1,950.60	75.02	2.96%
19	34	2,736.63	80.49	4.31%
20	18	1,404.97	78.05	1.95%
21	21	1,962.68	93.46	2.81%
22	20	2,089.21	104.46	3.44%
23	23	2,035.83	88.51	3.88%
24	15	913.07	60.87	1.47%
25	15	2,026.04	135.07	4.78%
26	19	1,376.88	72.47	2.65%
27	19	1,899.84	99.99	4.17%
28	14	1,402.74	100.20	3.05%
29	26	2,779.43	106.90	5.44%
Total	755	81,025.36	104.64	96.56%

Table S3. Different types of genes annotated by CNVRs in Pingliang red cattle genome.

Gene Type	No.of Genes	Percentage (%)
protein_coding	1,087	89.17%
lncRNA	62	5.09%
miRNA	24	1.97%
pseudogene	16	1.31%
snoRNA	12	0.98%
snRNA	5	0.41%
misc_RNA	3	0.25%
processed_pseudogene	3	0.25%
rRNA	2	0.16%
TR_V_gene	2	0.16%
scaRNA	1	0.08%
sRNA	1	0.08%
TR_J_gene	1	0.08%

Table S4. GO and KEGG pathway analyses of genes in the identified CNVRs.

Category	Term	GO Name	Count	*P*-value
GO_BP	GO:0036414	histone citrullination	5	1.18E-05
GO_BP	GO:0030036	actin cytoskeleton organization	13	2.48E-03
GO_BP	GO:0048167	regulation of synaptic plasticity	6	2.63E-03
GO_BP	GO:0015671	oxygen transport	4	2.98E-03
GO_BP	GO:0022604	regulation of cell morphogenesis	5	5.86E-03
GO_BP	GO:0007191	adenylate cyclase-activating dopamine receptor signaling pathway	4	6.01E-03
GO_BP	GO:2000311	regulation of AMPA receptor activity	5	7.10E-03
GO_BP	GO:0007010	cytoskeleton organization	9	8.73E-03
GO_BP	GO:0050808	synapse organization	6	1.31E-02
GO_BP	GO:0031532	actin cytoskeleton reorganization	7	1.36E-02
GO_BP	GO:0042744	hydrogen peroxide catabolic process	5	1.59E-02
GO_BP	GO:0030032	lamellipodium assembly	5	2.07E-02
GO_BP	GO:0050909	sensory perception of taste	4	2.35E-02
GO_BP	GO:0001771	immunological synapse formation	3	2.88E-02
GO_BP	GO:0007274	neuromuscular synaptic transmission	4	3.23E-02
GO_BP	GO:0034765	regulation of ion transmembrane transport	9	3.27E-02
GO_BP	GO:0043161	proteasome-mediated ubiquitin-dependent protein catabolic process	12	3.77E-02
GO_BP	GO:0001947	heart looping	6	4.04E-02
GO_BP	GO:0048856	anatomical structure development	6	4.36E-02
GO_BP	GO:0016055	Wnt signaling pathway	9	4.46E-02
GO_BP	GO:0098869	cellular oxidant detoxification	6	4.69E-02
GO_BP	GO:0016236	macroautophagy	4	4.83E-02
GO_BP	GO:0016540	protein autoprocessing	4	4.83E-02
GO_CC	GO:0031838	haptoglobin-hemoglobin complex	5	1.57E-03
GO_CC	GO:0005833	hemoglobin complex	5	2.07E-03
GO_CC	GO:0016323	basolateral plasma membrane	13	9.92E-03
GO_CC	GO:0005930	axoneme	9	1.31E-02
GO_CC	GO:0043197	dendritic spine	9	1.40E-02
GO_CC	GO:0015629	actin cytoskeleton	14	1.45E-02
GO_CC	GO:0098685	Schaffer collateral - CA1 synapse	7	1.97E-02
GO_CC	GO:0016020	membrane	44	2.38E-02
GO_CC	GO:0098978	glutamatergic synapse	15	3.17E-02
GO_CC	GO:1990454	L-type voltage-gated calcium channel complex	3	3.51E-02
GO_CC	GO:0030425	dendrite	16	3.74E-02
GO_CC	GO:0005815	microtubule organizing center	9	3.79E-02
GO_CC	GO:0009986	cell surface	22	3.84E-02
GO_CC	GO:0005737	cytoplasm	163	4.06E-02
GO_CC	GO:0005576	extracellular region	42	4.15E-02
GO_CC	GO:0031225	anchored component of membrane	6	4.16E-02
GO_MF	GO:0004930	G-protein coupled receptor activity	93	1.55E-09
GO_MF	GO:0004984	olfactory receptor activity	81	2.71E-09
GO_MF	GO:0004668	protein-arginine deiminase activity	5	1.06E-05
GO_MF	GO:0031721	hemoglobin alpha binding	5	1.35E-04
GO_MF	GO:0043177	organic acid binding	5	1.61E-03
GO_MF	GO:0005509	calcium ion binding	40	3.18E-03
GO_MF	GO:0016301	kinase activity	10	4.25E-03

GO_MF	GO:0005344	oxygen transporter activity	5	4.35E-03
GO_MF	GO:0030165	PDZ domain binding	8	6.69E-03
GO_MF	GO:0019825	oxygen binding	5	7.76E-03
GO_MF	GO:0004674	protein serine/threonine kinase activity	20	1.48E-02
GO_MF	GO:0005102	receptor binding	15	1.98E-02
GO_MF	GO:0005245	voltage-gated calcium channel activity	6	2.41E-02
GO_MF	GO:0005244	voltage-gated ion channel activity	5	3.02E-02
GO_MF	GO:0046872	metal ion binding	76	3.30E-02
GO_MF	GO:0005085	guanyl-nucleotide exchange factor activity	14	4.79E-02
KEGG_PATHWAY	bta04740	Olfactory transduction	84	7.73E-06
KEGG_PATHWAY	bta05133	Pertussis	10	6.77E-03
KEGG_PATHWAY	bta04917	Prolactin signaling pathway	10	1.09E-02
KEGG_PATHWAY	bta04658	Th1 and Th2 cell differentiation	11	1.11E-02
KEGG_PATHWAY	bta04510	Focal adhesion	16	3.04E-02
KEGG_PATHWAY	bta04010	MAPK signaling pathway	21	3.14E-02
KEGG_PATHWAY	bta04625	C-type lectin receptor signaling pathway	10	4.02E-02
KEGG_PATHWAY	bta01212	Fatty acid metabolism	7	4.03E-02
KEGG_PATHWAY	bta04971	Gastric acid secretion	8	4.80E-02

Table S5. Genes related to important economic traits annotated by CNVRs in Pingliang red cattle genome.

Category	Trait	Genes
Growth and development	Body depth	*KCNIP4, PAK5*
	Body weight	*CYP21, KCNIP4, CYLD*
	Feed conversion ratio	*CCKBR, PCCA, PRSS2*
	Net merit	*KCNIP4, MFGE8*
	Residual feed intake	*CCKBR, LRP5, PCCA, PRSS2*
	Rump width	*KCNIP4*
Carcass and meat quality	Carcass weight	*CACNA2D1, CYLD, NADK, TG, UBXN2B*
	Fat color	*CACNA2D1*
	Fat thickness at the 12th rib	*CACNA2D1, ITGA9*
	Intramuscular fat	*TG*
	Meat color	*TG*
	Muscle pH	*TG*
	Myristic acid content	*RAB11FIP3*
	Subcutaneous fat	*CDH12, GALNT13*
	Subcutaneous rump fat thickness	*SEMA5AA*
	Tenderness score	*ADGRV1*
Reproduction	Calving ease	*KCNIP4, MFGE8, PHACTR1*
	Daughter pregnancy rate	*ABCC9, MACROD2, NPFFR2, PAK5, TBC1D24, TRAPPC9*
	Early embryonic survival	*TBC1D24*
	Embryonic mortality	*ANXA10*
	Fertility index	*ACACA*
	First service conception	*KCNIP4, TBC1D24, TSGA10IP*
	Udder height	*KCNIP4, PAK5, SLC25A21*
Milk	305-day milk yield	*C4A, CACNA2D1*
	Milk fat percentage	*ABCC9, ADGRB1, APBA1, BAIAP2, C1R, C4A, CRACR2A, EPHA6, GPIHBP1, KCNIP4, KCNK9, LY6D, LY6E, MFGE8, MYO16, NNT, PLCE1, SCARB1, TG, TRAPPC9, ZNF696*
	Milk fat yield	*ABCC9, ADGRB1, ARAP1, ATF3, CRACR2A, CYP21, KCNIP4, KCNK9, LAMA4, LY6E, MACROD2, PAK5, SLC25A21, TRAPPC9, TRIM3, ZNF696*
	Milk yield	*ADGRB1, ATF3, BAIAP2, CACNA2D1, CRACR2A, DNAJC5, ERC2, FCHSD2, KCNIP4, KCNK9, LRP1, LY6E, NNT, NPFFR2, PAK5, SLC17A9, SLC25A21, TG, THSD7B, TPD52L2, TRAPPC9, ZBTB46, ZNF696*
	Milk arachidonic acid content	*FADS1*
	Milk beta-carotene content	*SCARB1*
	Milk beta-casein percentage	*SLC25A21*
	Milk C14 index	*PLCE1*
Exterior Traits	Duration of inactivity during open field test	*NFATC1*
	Temperament	*DPP3, MAP2K5, ZNF385D*

Table S6. Comparison of CNVR results obtained in this study with other studies conducted using various platforms.

Study	Platform	Breeds (Number*)	Sample	CNVR count	CNVR Length (Mb)	Overlapping CNVR count with present study(percentage)	Overlapping CNVR length with present study(percentage)
FADISTA et al., 2010[40]	CGH	Holstein, Simmental, etc. (4)	20	254	15.76	5(0.66%)	0.37(0.45%)
LIU et al., 2010[41]	CGH	Red Angus, Limousin, etc. (17)	90	163	25.06	6(0.79%)	0.75(0.93%)
ZHANG et al., 2015[42]	CGH	Jinnan, Luxi, etc. (12)	24	339	32.88	15(1.99%)	6.70(8.27%)
BICKHART et al., 2016[43]	CGH	Brahman, Nellore, etc. (8)	75	1853	87.50	73(9.67%)	16.46(20.32%)
JIANG et al., 2012[44]	50K	Chinese Holstein (1)	2047	96	23.90	2(0.26%)	0.14(0.17%)
BAE et al., 2010[45]	50K	Bos taurus coreanae (1)	265	368	62.70	24(3.18%)	7.50(9.26%)
CICCONARDI et al., 2013[46]	50K	Italian Friesian, Italian, Brown, etc. (5)	2654	402	498.00	25(3.31%)	7.48(9.23%)
GURGUL et al., 2015[21]	50K	Holstein, Polish Red (2)	1160	106	176.60	50(6.62%)	5.99(7.40%)
HUANG et al., 2017[47]	77K	Qinchuan (1)	254	255	184.00	53(7.02%)	7.01(8.65%)
JIANG et al., 2013[48]	BovineHD	Holstein (1)	96	367	42.74	27(3.58%)	7.10(8.76%)
UPADHYAY et al., 2017[49]	BovineHD	Dutch Friesian, Maltese, etc. (38)	149	923	61.06	37(4.90%)	10.58(13.06%)
SASAKI et al., 2016[50]	BovineHD	Japanese Black (1)	1481	861	43.65	44(5.83%)	12.42(15.33%)
JIA et al., 2019[8]	BovineHD	Polled yak (1)	215	1066	181.60	80(10.60%)	10.22(12.62%)
SILVA et al., 2016[9]	BovineHD	Nellore (1)	723	2649	170.60	116(15.36%)	21.70(26.78%)
XU et al., 2019[51]	BovineHD	Simmental (1)	1079	4912	248.70	182(24.11%)	30.57(37.73%)
ZHANG et al., 2020[22]	BovineHD	Aletai White, Anxi, etc. (26)	318	5818	379.95	208(27.55%)	27.99(34.55%)
ZHANG et al., 2015[52]	BovineHD	Qinchuan (1)	6	365	13.1	23(3.05%)	11.06(13.65%)
ZHOU et al., 2016[53]	BovineHD	Nellore (1)	2230	231	70.4	26(3.44%)	9.65(11.91%)
CHOI et al., 2013[23]	NGS	Hanwoo, Black Angus, etc. (3)	3	413-869	13-3.5	30(3.97%)	7.37(9.09%)
BICKHART et al., 2012[24]	NGS	Angus, Hereford, etc. (3)	5	1119	47.99	32(4.24%)	9.38(11.58%)
HU et al., 2020[26]	NGS	Nellore, Hereford, etc. (10)	73	13234	40.50	137(18.15%)	28.93(35.70%)
GAO et al., 2017[27]	NGS	Holstein (1)	8	6015	72.02	140(18.54%)	29.36(36.24%)
LIU et al., 2019[25]	NGS	Bubalus bubalis (1)	14	1344	59.8	50(6.62%)	14.86(18.34%)
This study	100K	Pingliang red (1)	122	755	81.03		

Note: * the number of breeds used for comparison.
